# Markers for the identification of tendon-derived stem cells in vitro and tendon stem cells in situ – update and future development

**DOI:** 10.1186/s13287-015-0097-y

**Published:** 2015-06-02

**Authors:** Pauline Po Yee Lui

**Affiliations:** Headquarter, Hospital Authority Building, 147B Argyle Street, Kowloon, Hong Kong, SAR China; Current address: 9/F, Rumsey Street, Multi-storey Carpark Building, 2 Rumsey Street, Sheung Wan, Hong Kong, SAR China

## Abstract

The efficacy of tendon-derived stem cells (TDSCs) for the promotion of tendon and tendon-bone junction repair has been reported in animal studies. Modulation of the tendon stem cell niche in vivo has also been reported to influence tendon structure. There is a need to have specific and reliable markers that can define TDSCs in vitro and tendon stem cells in situ for several reasons: to understand the basic biology of TDSCs and their subpopulations in vitro; to understand the identity, niches and functions of tendon/progenitor stem cells in vivo; to meet the governmental regulatory requirements for quality of TDSCs when translating the exciting preclinical findings into clinical trial/practice; and to develop new treatment strategies for mobilizing endogenous stem/progenitor cells in tendon. TDSCs were reported to express the common mesenchymal stem cell (MSC) markers and some embryonic stem cell (ESC) markers, and there were attempts to use these markers to label tendon stem cells in situ. Are these stem cell markers useful for the identification of TDSCs in vitro and tracking of tendon stem cells in situ? This review aims to discuss the values of the panel of MSC, ESC and tendon-related markers for the identification of TDSCs in vitro. Important factors influencing marker expression by TDSCs are discussed. The usefulness and limitations of the panel of MSC, ESC and tendon-related markers for tracking stem cells in tendon, especially tendon stem cells, in situ are then reviewed. Future research directions are proposed.

**Electronic supplementary material:**

The online version of this article (doi:10.1186/s13287-015-0097-y) contains supplementary material, which is available to authorized users.

## Introduction: importance of labeling tendon-derived stem cells in vitro and tracking stem cells in tendon in situ

The discovery of tendon stem/progenitor cells in the tendon mid-substance marks a new era for understanding the physiology and pathology of tendon as well as developing innovative therapeutics for the treatment of tendon and tendon-bone junction injuries [1]. Despite being heterogeneous cell populations, there is a huge need for markers to characterize and define the biological characteristics of tendon-derived stem cells (TDSCs) or their subpopulations in vitro, and to study the identity, niches and functions of stem/progenitor cells in tendon in vivo. This information is crucial for understanding the molecular/cellular mechanisms of tendon physiology and pathologies, and hence developing effective treatment strategies. Specific markers for quality control of TDSCs or their subpopulations are currently lacking, yet are crucial for the translation of research findings from bench to bed under current good manufacturing practice. Likewise, the functional modulation of stem/progenitor cells in tendon is an interesting approach to promote tendon and tendon-bone junction repair which may not require surgery, such as in mild acute tendon injury and chronic tendinopathy. The goal of modulating stem/progenitor cells in tendon is currently hampered by the limited data about their identity, niches and functions in tendon. In this review, I aim to update and discuss the future research directions of markers for defining TDSCs in vitro and stem cells in tendon, particularly tendon stem cells, in vivo. The terms ‘tendon-derived stem cells (TDSCs)’ and ‘stem cells in tendon’ refer to the stem/progenitor cells isolated from tendon mid-substances in vitro and detected in situ, respectively. The term ‘tendon stem cells’ refers to the stem/progenitor cells that reside in, and hence are specific to, tendon mid-substances.

## Markers characterizing tendon-derived stem cells in vitro

The Mesenchymal and Tissue Stem Cell Committee of the International Society for Cellular Therapy has proposed three minimal criteria to define human mesenchymal stem cells (MSCs). Among these criteria, more than 95 % of the isolated cells should express CD105, CD73 and CD90, and less than 2 % of the cells should express CD45, CD34, CD14 or CD11b, CD79a or CD19 and HLA-DR [2]. TDSCs hence meet the marker requirement of the International Society for Cellular Therapy for MSCs (Tables S1 and S2 in Additional file 1). They express CD90, CD73 and CD105 but are negative for CD31, CD34, CD45, HLA-DR, CD11b, CD14 and CD19 [3–5]. However, the International Society for Cellular Therapy’s proposed markers cannot uniquely distinguish TDSCs from other MSCs and some differentiated cells [6]. Many MSC markers are in fact fibroblast markers and the fibroblastic nature of MSCs, including TDSCs, may explain their expression in both fibroblasts and MSCs [7, 8]. Human skin or lung fibroblasts have been reported to express CD105, CD166, CD90, CD44, CD29, CD73 and CD9 as in human bone marrow-derived stem cells (BMSCs) [9]. Tendon explant culture that contained total tendon cells and mainly tenocytes expressed CD44, CD73 and CD90 at similar percentages to TDSCs, suggesting that these markers are not useful for quality control of TDSCs in cell-based therapies (unpublished observations). Rat tail tendon fibroblasts were also shown to express CD44 [10]. Ruzzini and colleagues prospectively purified CD44^+^ cells from human semitendinosus tendons and reported that they were stem cells, and speculated that the CD44^–^ cells were tenocytes because they did not express CD146 and Stro-1 [11]. However, further study on the colony-forming ability and multilineage differentiation potential of the tendon fibroblasts in these two studies is needed [10, 11]. Some information about the CD markers’ functions is available although their exact functions in stem cells are poorly understood [12–14]. The crude definition of MSCs based only on in vitro assays creates difficulties in identifying specific markers for *bona fide* stem cells [15]. Better understanding of the in vivo functions of MSC markers in TDSCs will rationalize and facilitate the selection of appropriate markers for distinguishing TDSCs from tenocytes.

Collagen type I, collagen type III, tenascin C, scleraxis (Scx) and tenomodulin (Tnmd) are commonly used tendon-related markers. They alone are also not useful for distinguishing TDSCs from tenocytes because both cell types express these markers. Stemness is not a frozen condition but a continuous process. Hence we do not know the point at which the expression of tenogenic markers alone is associated with the loss of self-renewal and multipotency – the definition of terminal differentiation. TDSCs expressed very high levels of *Scx*, *Tnmd* and Mohawk (*Mkx*) [16, 17]. Contrary to expectation, the loss of Tnmd resulted in reduced clonogenicity and proliferative potential as well as earlier and higher incidence of cellular senescence, but has no profound effect on the in vitro multilineage differentiation potential of mouse TDSCs [18]. Ectopic expression of *Tnmd* in wild-type mouse TDSCs increased cell proliferation [18]. This apparently contradicts the use of Tnmd as a tenogenic differentiation maker and hence more sophisticated regulation of Tnmd in stem cells is in place. Whether the loss of Scx is associated with increased or loss of stemness of TDSCs requires further research. However, overexpression of *Scx* has been associated with the loss of colony-forming ability, proliferation and in vitro multilineage differentiation potential of an immortalized BMSC line [19]. Ectopic expression of *Scx* combined with force has also been reported to synergistically promote the commitment of MSCs derived from human embryonic stem cells (ESCs) to tenocytes [20]. Ectopic *Mkx* expression has been reported to promote lineage commitment and in vitro tenogenesis more efficiently than *Scx* in a C3H10T1/2 mouse MSC line [17]. Overexpression of *Mkx* also enhanced expression of Scx protein and mRNA, while silencing of *Mkx* downregulated *Scx*, decorin (*Dcn*) and *Tnmd* mRNA expression in mouse tail TDSCs [17]. Early growth response (Egr)1 was reported to enhance the expression of tendon-related markers and to inhibit in vitro nontenocyte differentiation of rabbit TDSCs [21].

TDSCs have been reported to express Oct-4, Nanog, nucleostemin, SSEA-4, c-myc and Sox2 [22–26] (Table S1 in Additional file 1). These ESC markers might be useful for distinguishing TDSCs from tenocytes because a previous study has shown that tendon cells isolated from culture after removal of TDSC colonies presented limited in vitro multilineage differentiation potential and did not express Oct-4, SSEA-4 and nucleostemin [26]. There has been no study directly comparing the expression of these ESC markers in TDSCs with the truly pluripotent cells. Information demonstrating the causal roles of these ESC markers in the self-renewal and multipotency of TDSCs is also lacking. Further research in these areas is required. Some ESC markers including Oct-4, Nanog, nucleostemin and Sox2 are nuclear proteins that limit their use as markers for prospective isolation of TDSCs in clinical trials/practices. Surface markers such as the SSEA families, if shown to be valid, might be more useful for the prospective isolation of TDSCs.

## Factors influencing marker expression by tendon-derived stem cells

There are discrepancies in the expression of some markers such as CD71, CD146, Stro-1, CD105 and CD166 by TDSCs in different studies (Table S1 in Additional file 1). Marker expression in TDSCs can be influenced by the cell source, procedures of cell isolation and cell culture.

### Cell source

#### Age

The age of the animal or human may affect the properties, including marker expression, of TDSCs. While nearly 100 % of young and aged rat TDSCs expressed nucleostemin, Oct-4 and SSEA-4, the aged TDSCs expressed a lower percentage and cellular expression of CD90.1 but a higher percentage and cellular expression of CD44 compared with young cells [25]. On the other hand, Kohler and colleagues reported no difference in the expression of CD73, CD90, CD105, STRO-1, CD146 and Musashi-1 in young and healthy TDSCs compared with aged and degenerated TDSCs isolated from human nonruptured Achilles tendons [27]. Ruzzini and colleagues did not report any difference in the expression of CD146 and STRO-1 with age in CD44^+^ TDSCs purified from healthy human semitendinosus tendon graft [11]. However, the sample sizes in both of these studies were small: *n* = 3 [27] and *n* = 2 [11] per age group. Further research is needed to confirm the effect of age on the properties, including marker expression, of TDSCs.

#### Donors

The growth potential and immunophenotype of mouse BMSCs have been reported to vary between strains [28]. Systematic study of the species-specific differences of marker expression in TDSCs is lacking. The current limited research, however, has not identified a marker that is exclusively present or absent in some species (Tables S1 and S2 in Additional file 1). Markers that are expressed across different species may have the highest translational value.

#### Tendon types

TDSCs isolated from different tendons may exhibit differences in marker expression and hence functions. Dyment and colleagues reported that patellar tendon was the only tendon that has a mixture of both tendon and ligament marker expression [29]. The generalization of research findings (including TDSC markers, which are the subject of this review) generated from patellar tendon to other tendons therefore needs to be cautious. Zhang and Wang reported that both patellar and Achilles TDSCs expressed Oct-4, SSEA-4 and nucleostemin without mentioning any differences in the expression levels between these two cell sources [26]. More systematic investigation into the similarities and differences of TDSCs isolated from different tendons are needed.

### Procedures of cell isolation

#### Contamination by stem/progenitor cells in neighboring tissues

Contamination by stem/progenitor cells present in nearby tissues is possible as TDSCs are isolated by enrichment. This is especially an issue when the source tendon is degenerated or injured. Nonhematopoietic adult stem cells of different mesenchymal tissues probably express a similar, although nonidentical, set of MSC and ESC markers. They express some common markers because they share fibroblast characteristics (see Markers characterizing tendon-derived stem cells in vitro) and common functions of stem cells. This implies that the use of common MSC and ESC markers which are not specifically gauged to the tissue-specific functions of stem cells cannot distinguish TDSCs from other MSCs.

A recent study has shown that mouse stem/progenitors isolated from the peritenon and tendon proper were Sca-1^+^, CD90.2^+^, CD44^+^, CD18^–^ and CD133^–^ [30], and hence these markers cannot be used to distinguish them. Another study has reported that peritenon cells isolated from horse superficial digital flexor tendon expressed higher mRNA level of *cd45* (CD45), *Thy1* (CD90), *Eng* (CD105) and *Oct4* as well as lower mRNA and protein levels of *Scx* compared with cells isolated from the tendon proper [31]. Reparative mesenchymal cells have been reported entering tendon after injury via blood circulation [32]. P75^+^ neural-crest-like stem/progenitor cells of perivascular origin have been reported to reside within the peritenon and give rise to scar tissues following patellar tendon injury in rats [33]. Another study has also reported the migration of SMA^+^ cells in the peritenon to the tendon injury site in the mid-substance, contributing to tendon repair [29].

It is hypothesized that healing mediated by extraneous cell populations (extrinsic healing) forms scar tissues and adhesions while healing mediated by endogenous cells (intrinsic healing) promotes tendon regeneration [31, 34]. The ability to distinguish tendon stem/progenitor cells from circulation-derived stem/progenitor cells and neighboring tissues, particularly peritenon and tendon–bone junction, is therefore important. TDSCs showed higher mRNA expression of *Tnmd*, *Scx* and *Mkx* compared with other stem cells [16, 17, 30, 35]. A recent study has reported that tendon proper-derived progenitors expressed higher mRNA levels of *Scx* and trends for *Tnmd* but a lower mRNA level of *Emcn* (a vascular marker) compared with peritenon-derived progenitors [36]. They have further shown that Scx protein (as shown by green fluorescence protein (GFP) reporter) was expressed in all cells in the tendon proper but not in the cells of the surrounding peritenon of Achilles tendon of 1-day-old mouse [36]. A combination of tendon-related markers and stem cell-specific markers (some ESC markers mentioned above may be used as a start for testing) may be able to distinguish TDSCs from other MSCs and tenocytes.

Until specific TDSC markers are identified, the purity of tissue used for TDSC isolation is critical for quality control. The use of tendon from an injured or diseased patient or a healthy donor for TDSC isolation is also critical. Understanding the relationship of the potential markers with the in vivo tendon-specific functions of stem cells is essential for the identification of specific markers distinguishing TDSCs from other MSCs.

#### Method of tissue digestion

The method of tissue digestion can potentially affect the retention of antigenicity and the analysis of surface markers [37]. Panchision and colleagues have reported that CD24 antigenicity, which was retained after Liberase-1 (a collagenase and neutral protease cocktail) and TrypLE (a recombinant trypsin-like replacement) treatment, provided a useful discrimination of mouse fetal multipotent stem cells from neuronal-committed progenitors and post-mitotic neurons when paired with CD133 or CD15 [37]. However, this particular discrimination of the two interesting cell subpopulations was not possible after papain treatment of neural tissues because the expression of CD24 was lost. Some research groups have used dispase in addition to collagenase type I [3, 26], while other groups have used collagenase type II [27, 38], protease type XIV followed by collagenase B [31] or collagenase type V [5] for tendon digestion for TDSC isolation. The influence of the method of tissue digestion on the subsequent surface marker analysis requires further research.

### Procedures of cell culture

#### Effect of culture conditions

Culture conditions can influence phenotypes, including marker expression of TDSCs. Growth factors in fetal calf serum, culture confluency, the topography and material of the cell culture surface, mechanical loading and oxygen tension can induce or suppress marker expression. Human TDSCs cultured under 5 % oxygen expressed higher levels of nucleostemin, Oct-4, Nanog and SSEA-4 as shown by immunohistochemistry and/or quantitative RT-PCR [39]. Another study has shown that the surface expression of CD44, CD73, CD90 and CD105 in human TDSCs was maintained under hypoxia (2 % oxygen) as shown by flow cytometry [40]. 2-Mercaptoethanol was added to the initial cell culture for TDSC isolation in some studies [3, 11, 30]. 2-Mercaptoethanol has been reported to improve the culture environment by reducing reactive oxygen species and to promote cell growth by reducing cystine to cysteine needed for cell growth [41]. However, both 2-mercaptoethanol and cysteine have been reported in other studies as inducing agents for neuronal-like cell differentiation in vitro [42, 43]. A recent study has isolated neural crest-like stem/progenitor cells from rat patellar tendon mid-substance by culturing tendon-derived cells in an optimized neural crest stem cell medium containing 2-mercaptoethanol [33]. The isolated cells expressed a panel of neural crest stem cell markers (P75, vimentin, Snail, Sox10) in addition to CD29 and CD90 [33]. They could also differentiate into both mesenchymal (osteogenic, chondrogenic, adipogenic and myogenic) and neural lineages in vitro upon induction [33]. The use of neural crest stem cell medium to culture tendon-derived cells might create some selective pressure on cells and change their fate commitment. There is currently no systematic study of the effect of 2-mercaptoethanol on the biological characteristics including marker expression of TDSCs.

#### Effect of cell passaging

The biological characteristics of many cell types change after isolation from the in vivo environment and in vitro passaging. The surface expression of CD90 and CD73 was downregulated during in vitro passaging of rat patellar TDSCs [44]. CD146 was expressed in some freshly isolated TDSCs but the expression was lost after passaging [24], which explains the absence of CD146 in TDSCs in our earlier study [40]. The use of early and consistent cell passages for experiments and trials is therefore recommended for reproducible outcomes [44].

## Markers for labeling tendon stem cells in situ

Many of the cues about the native frequency, niches and functions of tendon stem cells are derived from TDSCs which are defined and characterized in vitro. Most in vitro markers are aimed originally to prospectively enrich the stem cell subset endowed with clonogenicity, not to identify where the cells originated in the tissue. The possibility exists that the phenotypes and functions of TDSCs and tendon stem cells in vivo differ due to the isolation of TDSCs from their native environment and culture conditions that may alter their characteristics. In contrast to the previous finding of high CD44 expression on culture-expanded MSCs, Qian and colleagues found that BMSCs physiologically did not express CD44 [45]. In vitro culture could result in the acquisition of CD44 expression and changes in the expression of cytokines, growth factors, matrix proteins and other signaling molecules [45]. While CD44 and Sca-1 are used as in vitro stem cell markers of TDSCs, nearly all of the cells in rat patellar tendon were labeled in situ [24] and hence they are not good in vivo markers. One study has reported the labeling of stem cells in rat Achilles tendon in situ by immunohistochemical staining of nucleostemin, and the density of nucleostemin-positive cells was twice as high at the tendon–calcaneus junction compared with that at the tendon mid-substance [46]. However, another study has shown that nucleostemin and other ESC markers (Oct-4, Nanog and Sox2) were absent in all cells, including the iododeoxyuridine (IdU) label-retaining cells, in intact rat patellar tendon as shown by immunohistochemical staining [24]. However, these ESC markers were expressed in most of the label-retaining cells in injured tendon in vivo and in TDSCs in vitro [24], suggesting that the developmental program might be reactivated during tendon injury. Caution has to be taken when extrapolating the in vitro findings to in vivo conditions.

Because the existing in vitro markers do not work well in vivo, other methods are sought to track the stem cells in vivo. Prelabeling of a specific cell population in vivo with true stemness markers or lineage-specific markers, combined with spatial information as provided by histology or microdissection as well as more frequent follow-up (sampling), may help to dissect the cell sources and mechanisms for tendon repair. Using the IdU labeling-retaining method, Bi and colleagues [3] and Tan and colleagues [24] tried to label the stem cells in tendon in situ. One potential limitation of the IdU label-retaining method is that it is not specific for tendon stem cells and all stem cells in the body are labeled. The sources of stem cells that contribute to different physiological or pathological processes in tendon cannot be distinguished.

Beside the IdU label-retaining method, the detection of telomerase-positive cells [47] and genetic-based lineage tracing using tissue-specific developmental proteins [48] may be useful for tracing stem cells in general and tendon stem cells, respectively. Developmental markers including Sox and sonic hedgehog have been used for tracing skin progenitor cells [49] and post-mitotic precursors of taste cells [50], respectively, in situ. Tnmd and Scx are important proteins for tendon development [51, 52]. Both the active form of Smad8, in the presence of BMP2, and Scx have been reported to drive the tenogenic reprogramming of immortalized BMSC lines [19, 53]. However, most cells in intact adult tendon expressed Scx, Tnmd and Smad8 [24], making it impossible to use these markers to track the fate of tendon stem cells in situ. Whether tendon developmental proteins including Mkx, Egr1, Egr2, Eya1, Eya2, Six1, Six2, Epha4 and Thbs4 can be used for tracing tendon stem cells in situ requires further research.

Using the genetic-based lineage tracing technique, Sox9-expessing precursors have been reported as the cellular origin for cruciate ligament of the knee joint and limb tendons [54]. Using a similar lineage tracing technique for tracking Sox9^+^ progenitors, another study has reported that tendon cells were derived from Scx^+^/Sox9^+^ and Scx^+^/Sox9^–^ progenitors [55]. The closer the tendon was to the cartilaginous junction, the more tendon cells arose from the Scx^+^/Sox9^+^ progenitors [55]. Dyment and colleagues used an inducible Cre driven by alpha-smooth muscle actin (α-SMA, a marker for vascular muscle cells and myofibroblasts) and a constitutively active Cre driven by growth differentiation factor-5 (GDF5, a chondrogenic and tenogenic factor), in combination with a Cre reporter to trace SMA^+^ and GDF5^+^ populations and their progeny in mice [29]. They reported that SMA^+^ cells were found in the peritenon, as well as in the Scx^+^ cells within tendon mid-substance and myotendinous junction, but they were not found in tendon entheses or knee ligaments. In contrast, GDF5^+^ cells were found in tendon entheses and knee ligaments. Using the SMA^+^ lineage marker, they have reported that SMA^+^ cells migrated to the tendon injury site in the mid-substance and differentiated into Scx^+^ cells [29]. Further studies are required to confirm that the isolated SMA^+^ population, including the SMA^+^Scx^+^ population in tendon, and the GDF5^+^ population are stem/progenitor cells by showing their self-renewal and multilineage differentiation potential and by showing their effects on tendon development with conditional inactivation/activation. Tracking of the lineage of hedgehog-responsive cells based on Gli1 expression showed that a unique population of hedgehog-responsive cells originated from the developing enthesis was necessary for fibrocartilage progenitor cell differentiation and eventually fibrocartilage mineralization in the enthesis in adults [56]. Similar to the IdU label-retaining method, the detection of telomerase activity is not specific to tendon stem cells. The genetic-based lineage tracing method has the advantage of being more specific if useful developmental markers can be found.

The combined use of transgenic animals with cells labeled in different colors and cell transplantation may facilitate the tracking of specific stem/progenitor cell populations in situ, although exogenous cells are used and hence this may raise concerns about their relevance to the understanding of the physiological and natural healing or injury processes. Using the CD-1 mouse model, the CD-1 nude mouse model, the red fluorescent protein transgenic mouse model, the GFP transgenic mouse model, the Scx–GFP reporter mouse model and the compound Scx–GFP reporter–red fluorescent protein mouse model, Asai and colleagues have shown that two populations of stem/progenitor cells isolated from injured tendons had distinct potentials to participate in tendon regeneration and chondroid degeneration after transplantation to the injury site, although the origins of these cells were not elucidated in the study [35].

## Future research directions

### Understanding in vivo functions of common MSC and ESC markers of tendon-derived stem cells

Markers defining the properties of *bona fide* stem cells can only be identified through the use of in vivo assays which functionally define stem cells [15]. While TDSCs expressed many of the common MSC markers and some ESC markers, the exact functions of these markers in TDSCs are still poorly understood. Better understanding of their in vivo functions using overexpression and knockdown approaches as well as transplantation in null mouse models are important to better select markers associated with stemness (that is, self-renewal and multipotency) of TDSCs.

### High-throughput technologies for distinguishing tendon-derived stem cells from nonhematopoietic adult stem cells of other mesenchymal tissues and tenocytes

High-throughput technologies such as single-cell genome analysis and transcriptome analysis of cultures of TDSCs, tenocytes and nonhematopoietic adult stem cells of other mesenchymal tissues can be used to search for correlated gene clusters to classify cell types, dissect cell heterogeneity, map out cellular hierarchy and construct genetic networks [57–61]. Using a single-cell quantitative PCR technique, Guo and colleagues have designed an analysis platform to cover all commonly used cell surface markers with a total of 280 genes for all mouse cell types [57]. Hierarchical clustering of the single-cell data faithfully grouped stem cells of the same origin together and revealed some lineage-specific markers. Using the same platform, the authors further analyzed the gene expression in all key cell populations of the mouse hematopoietic system and hierarchically mapped out the mouse hematopoietic system and hematopoietic stem cell differentiation stages [57]. The clustering pattern may then be used to identify novel markers and populations [57].

Using genome-wide gene expression analysis, Kaltz and colleagues have shown that BMSCs are distinct from umbilical vein stromal cells [59]. Novel membrane-associated markers were identified in BMSCs when compared with umbilical vein stromal cells [59]. Mathematical models based on DNA methylation of biomarkers have been reported to distinguish induced pluripotent stem cells, ESCs and somatic cells with high accuracy [61]. High-throughput cell surface antigen profiling using an antibody array may also be useful for the identification of specific TDSC surface markers [62, 63]. In all cases, confirmation of the in vivo functions of potential markers identified by these high-throughput technologies is needed.

### Characterization of tendon-derived stem cell subpopulations with coexpressed markers

As heterogeneous cell populations, the identification of a single marker that can specifically define TDSCs is expected to be unfeasible. The current use of multiple single markers for their identification therefore has limitations because the markers may not be coexpressed by the same cell. The characterization of TDSCs and their subpopulations using hierarchical and coexpressed markers should be one research direction. Flow cytometry supports the simultaneous detection of multiple markers, which facilitates TDSC characterization and quality control. Flow cytometric analysis of marker expression in stem cells can be challenging as there is often no clear distinction between positive and negative populations [64]. Much attention therefore needs to be put on the correct gating during flow cytometric analysis. Tormin and colleagues sorted BMSCs based on their proliferation characteristics and identified surface markers for the characterization of these BMSC subpopulations by gene expression profiling [65]. Gauged by the Sca-1-positive signal, the CD105^+^ and CD105^–^ subpopulations of stem/progenitor cells were found to show higher potentials in tendon regeneration and chondroid degeneration, respectively, after transplantation in the injured mouse Achilles tendon [35].

### Labeling tendon stem cells in situ

Better understanding of the tendon induction and differentiation during development would help the discovery of specific markers for lineage tracing [66, 67]. The study of the usefulness of tendon developmental proteins including Mkx, Egr1, Egr2, Eya1, Eya2, Six1, Six2, Epha4 and Thb4 for tracing endogenous tendon stem cells should be one research direction. Methods such as the IdU label-retaining method, staining for telomerase and measurement of telomerase activity are not specific for labeling tendon stem cells but are useful for distinguishing stem cells from terminally differentiated cells. When combined with the spatial information in histology and immunohistochemical staining of tendon-related markers such as Scx and Tnmd, these methods are useful for tracing stem cells in tendon and may have the chance of distinguishing different stem cell populations. Given that the technique’s limitations are recognized, the combined use of transgenic animals with cells labeled in different colors and cell transplantation may also be useful for tracing stem/progenitor cells in situ.

## Conclusion

There is a huge need for specific and reliable markers that can label TDSCs in vitro and tendon stem cells in situ. Many of the existing markers seen as defining MSCs are simply widely shared and expressed by fibroblasts, and hence these markers do not imply any true in vivo stem cell property that defines true stem cells. Better understanding of in vivo functions of common MSC and ESC markers is important for identifying markers associated with stemness (that is, self-renewal and multipotency) of TDSCs. High-throughput gene or protein profiling may be useful for identifying markers that can distinguish TDSCs from other cells. The characterization of TDSCs and their subpopulations using hierarchical markers should be one research direction. Researchers should be aware of the influence of cell source, procedures of cell isolation and cell culture on marker expression and hence the functions of TDSCs. Standardization of TDSC and tenocyte culture in different laboratories will facilitate the identification of TDSC-specific markers. Markers that are used for the identification of TDSCs in vitro may not be useful for tracking of tendon stem cells in situ. Studies on tendon development might help in the discovery of specific markers for tracing tendon stem cells in situ.

## Additional file

Additional file 1:
**Contains Table S1 and Table S2 presenting protein expression of positive and negative markers in TDSCs isolated from different tendons in different species.**

## Abbreviations

BMSC: Bone marrow-derived stem cell
Egr: Early growth response
ESC: Embryonic stem cell
GDF5: Growth differentiation factor 5
GFP: Green fluorescence protein
IdU: Iododeoxyuridine
Mkx: Mohawk
MSC: Mesenchymal stem cell
Scx: Scleraxis
SMA: Smooth muscle actin
TDSC: Tendon-derived stem cell
Tnmd: Tenomodulin

## References

[1] Lui PP, Chan KM (2011). Tendon-derived stem cells (TDSCs): from basic science to potential roles in tendon pathology and tissue engineering applications. Stem Cell Rev..

[2] Dominici M, Le Blanc K, Mueller I, Slaper-Cortenbach I, Marini F, Krause D (2006). Minimal criteria for defining multipotent mesenchymal stromal cells. The International Society for Cellular Therapy position statement. Cytotherapy..

[3] Bi Y, Ehirchiou D, Kilts TM, Inkson CA, Embree MC, Sonoyama W (2007). Identification of tendon stem/progenitor cells and the role of the extracellular matrix in their niche. Nat Med..

[4] Randelli P, Conforti E, Piccoli M, Ragone V, Creo P, Cirillo F (2013). Isolation and characterization of 2 new human rotator cuff and long head of biceps tendon cells possessing stem cell-like self-renewal and multipotential differentiation capacity. Am J Sports Med..

[5] Utsunomiya H, Uchida S, Sekiya I, Sakai A, Moridera K, Nakamura T (2013). Isolation and characterization of human mesenchymal stem cells derived from shoulder tissues involved in rotator cuff tears. Am J Sports Med..

[6] Alt E, Yan Y, Gehmert S, Song YH, Altman A, Gehmert S (2011). Fibroblasts share mesenchymal phenotypes with stem cells, but lack their differentiation and colony-forming potential. Biol Cell..

[7] Haniffa MA, Collin MP, Buckley CD, Dazzi F (2009). Mesenchymal stem cells: the fibroblasts’ new clothes?. Haematologica..

[8] Hematti P (2012). Mesenchymal stromal cells and fibroblasts: a case of mistaken identity?. Cytotherapy..

[9] Halfon S, Abramov N, Grinblat B, Ginis I (2011). Markers distinguishing mesenchymal stem cells from fibroblasts are downregulated with passaging. Stem Cells Dev..

[10] Crockett RJ, Centrella M, McCarthy TL, Grant TJ (2010). Effects of cyclic strain on rat tail tenocytes. Mol Biol Rep..

[11] Ruzzini L, Abbruzzese F, Rainer A, Longo UG, Trombettta M, Maffulli N (2014). Characterization of age-related changes of tendon stem cells from adult human tendons. Knee Surg Sports Traumatol Arthrosc..

[12] Affymetrix eBioscience. Human CD & other cellular antigens – antibodies for multicolor flow cytometry, functional assays and immunohistochemistry. http://www.ebioscience.com/resources/human-cd-chart.htm. Accessed 7 Jul 2014.

[13] Affymetrix eBioscience. Mouse CD & other cellular antigens – antibodies for multicolor flow cytometry, functional assays and immunohistochemistry. http://www.ebioscience.com/resources/mouse-cd-chart.htm. Accessed 7 Jul 2014.

[14] BD Biosciences. CD marker handbook. https://www.bdbiosciences.com/documents/cd_marker_handbook.pdf. Accessed 7 Jul 2014.

[15] Bianco P, Robey PG, Simmons PJ (2008). Mesenchymal stem cells: revisiting history, concepts and assays. Cell Stem Cell..

[16] Tan Q, Lui PPY, Rui YF, Wong YM (2012). Comparison of potentials of stem cells isolated from tendon and bone marrow for musculoskeletal tissue engineering. Tissue Eng Part A..

[17] Liu H, Zhang C, Zhu S, Lu P, Zhu T, Gong X (2015). Mohawk promotes the tenogenesis of mesenchymal stem cells through activation of the TGFβ signaling pathway. Stem Cells..

[18] Alberton P, Dex S, Popov C, Shukunami C, Schieker M, Docheva D (2015). Loss of tenomodulin results in reduced self-renewal and augmented senescence of tendon stem/progenitor cells. Stem Cells Dev..

[19] Alberton P, Popov C, Pragert M, Kohler J, Shukunami C, Schieker M (2012). Conversion of human bone marrow-derived mesenchymal stem cells into tendon progenitor cells by ectopic expression of scleraxis. Stem Cells Dev..

[20] Chen X, Yin Z, Chen JL, Shen WL, Liu HH, Tang QM (2012). Force and scleraxis synergistically promote the commitment of human ES cells derived MSCs to tenocytes. Sci Rep..

[21] Tao X, Liu J, Chen L, Zhou Y, Tang K (2015). EGR1 induces tenogenic differentiation of tendon stem cells and promotes rabbit rotator cuff repair. Cell Physiol Biochem..

[22] Jiang D, Xu B, Yang M, Zhao Z, Zhang Y, Li Z (2014). Efficacy of tendon stem cells in fibroblast-derived matrix for tendon tissue engineering. Cytotherapy..

[23] Lovati AB, Corradetti B, Lange Consiglio A, Recordati C, Bonacina E, Bizzaro D (2011). Characterization and differentiation of equine tendon-derived progenitor cells. J Biol Regul Homeost Agents..

[24] Tan Q, Lui PP, Lee YW (2013). In vivo identity of tendon stem cells and the roles of stem cells in tendon healing. Stem Cell Dev..

[25] Zhou Z, Akinbiyi T, Xu L, Ramcharan M, Leong DJ, Ros SJ (2010). Tendon-derived stem/progenitor cell aging: defective self-renewal and altered fate. Aging Cell..

[26] Zhang J, Wang JHC (2010). Characterization of differential properties of rabbit tendon stem cells and tenocytes. BMC Musculoskelet Disord..

[27] Kohler J, Popov C, Klotz B, Alberton P, Prall WC, Haasters F (2013). Uncovering the cellular and molecular changes in tendon stem/progenitor cells attributed to tendon aging and degeneration. Aging Cell..

[28] Peister A, Mellad JA, Larson BL, Hall BM, Gibson LF, Prockop DJ (2004). Adult stem cells from bone marrow (MSCs) isolated from different strains of inbred mice vary in surface epitopes, rates of proliferation, and differential potential. Blood..

[29] Dyment NA, Hagiwara Y, Matthews BG, Li Y, Kalajzic I, Rowe DW (2014). Lineage tracing of resident tendon progenitor cells during growth and natural healing. PLoS One..

[30] Mienaltowski MJ, Adams SM, Birk DE (2013). Regional differences in stem cell/progenitor cell populations from the mouse Achilles tendon. Tissue Eng Part A..

[31] Cadby JA, Buehler E, Godbout C, van Weeren PR, Snedeker JG (2014). Differences between the cell populations from the peritenon and the tendon core with regard to their potential implication in tendon repair. PLoS One..

[32] Kajikawa Y, Morihara T, Watanabe N, Sakamoto H, Matsuda K, Kobayashi M (2007). GFP chimeric models exhibited a biphasic pattern of mesenchymal cell invasion in tendon healing. J Cell Physiol..

[33] Xu W, Sun Y, Zhang J, Xu K, Pan L, He L (2015). Perivascular derived stem cells with neural crest characteristics are involved in tendon repair. Stem Cell Dev..

[34] Khan U, Occleston NL, Khaw PT, McGrouther DA (1997). Single exposures to 5-fluorouracil: a possible mode of targeted therapy to reduce contractile scarring in the injury tendon. Plast Reconstr Surg..

[35] Asai S, Otsuru S, Candela ME, Cantley L, Uchibe K, Hofmann TJ (2014). Tendon progenitor cells in injured tendons have strong chondrogenic potential: the CD105-negative subpopulation induces chondrogenic degeneration. Stem Cells..

[36] Mienaltowski MJ, Adams SM, Birk DE (2014). Tendon proper- and peritenon-derived progenitor cells have unique tenogenic properties. Stem Cells Res Ther..

[37] Panchision DM, Chen HL, Pistollato F, Papini D, Ni HT, Hawley TS (2007). Optimized flow cytometric analysis of central nervous system tissue reveals novel functional relationships among cells expressing CD133, CD15, and CD24. Stem Cells..

[38] Haasters F, Polzer H, Prall WC, Saller MM, Kohler J, Grote S (2011). Bupivacaine, ropivacaine, and morphine: comparison of toxicity on human hamstring-derived stem/progenitor cells. Knee Surg Sports Traumatol Arthrosc..

[39] Zhang J, Wang JH (2013). Human tendon stem cells better maintain their stemness in hypoxic culture conditions. PLoS One..

[40] Lee WYW, Lui PP, Rui YF (2012). Hypoxia mediated efficient expansion of human tendon-derived stem cells (hTDSCs) in vitro. Tissue Eng Part A..

[41] Bannai S (1992). Use of 2-mercaptoethanol in cell culture. Hum Cell..

[42] Soleimani Mehranjani M, Chian MF (2014). Cysteine: a novel neural inducer for rat bone marrow mesenchymal stem cells. Cell J..

[43] Woodbury D, Schwarz EJ, Prockop DJ, Black IB (2000). Adult rat and human bone marrow stromal cells differentiate into neurons. J Neurosci Res..

[44] Tan Q, Lui PP, Rui YF (2012). Effect of in vitro passaging on the stem cell-related properties of tendon-derived stem cells – implications in tissue engineering. Stem Cell Dev..

[45] Qian H, Le Blanc K, Sigvardsson M (2012). Primary mesenchymal stem and progenitor cells from bone marrow lack expression of CD44 protein. J Biol Chem..

[46] Runesson E, Ackermann P, Brisby H, Karlsson J, Eriksson BI (2013). Detection of slow-cycling and stem/progenitor cells in different regions of rat Achilles tendon: response to treadmill exercise. Knee Surg Sports Traumatol Arthrosc..

[47] Breault DT, Min IM, Carlone DL, Farilla LG, Ambruzs DM, Henderson DE (2008). Generation of mTert-GFP mice as a model to identify and study tissue progenitor cells. Proc Natl Acad Sci U S A..

[48] Speer MY, Yang HY, Brabb T, Leaf E, Look A, Lin WL (2009). Smooth muscle cells give rise to osteochondrogenic precursors and chondrocytes in calcifying arteries. Circ Res..

[49] Liu S, Herault Y, Pavlovic G, Leask A (2014). Skin progenitor cells contribute to bleomycin-induced skin fibrosis. Arthritis Rheumatol..

[50] Miura H, Scott JK, Harada S, Barlow LA (2014). Sonic hedgehog-expressing basal cells are general post-mitotic precursors of functional taste receptors cells. Dev Dyn..

[51] Docheva D, Hunziker EB, Fassler R, Brandau O (2005). Tenomodulin is necessary for tenocyte proliferation and tendon maturation. Mol Cell Biol..

[52] Schweitzer R, Chyung JH, Murtaugh LC, Brent AE, Rosen V, Olson EN (2001). Analysis of the tendon cell fate using Scleraxis, a specific marker for tendons and ligaments. Development..

[53] Hoffmann A, Pelled G, Turgeman G, Eberle P, Zilberman Y, Shinar H (2006). Neotendon formation induced by manipulation of the Smad8 signaling pathway in mesenchymal stem cells. J Clin Invest..

[54] Soeda T, Deng JM, de Crombrugghe B, Behringer RR, Nakamura T, Akiyama H (2010). Sox9-expressing precursors are the cellular origin of the cruciate ligament of the knee joint and the limb tendons. Genesis..

[55] Sugimoto Y, Takimoto A, Akiyama H, Kist R, Scherer G, Nakamura T (2013). Scx+/Sox9+ progenitors contribute to the establishment of the junction between cartilage and tendon/ligament. Development..

[56] Schwartz AG, Long F, Thomopoulos S (2015). Enthesis fibrocartilage cells originate from a population of Hedgehog-responsive cells modulated by the loading environment. Development..

[57] Guo G, Luc S, Marco E, Lin TW, Peng C, Kerenyi MA (2013). Mapping cellular hierarchy by single-cell analysis of the cell surface repertoire. Cell Stem Cell..

[58] Bae S, Ah JH, Park CW, Son HK, Kim KS, Lim NK (2009). Gene and microRNA expression signatures of human mesenchymal stromal cells in comparison to fibroblasts. Cell Tissue Res..

[59] Kaltz N, Ringe J, Holzwarth C, Charbord P, Niemeyer M, Jacobs VR (2010). Novel markers of mesenchymal stem cells defined by genome-wide expression analysis of stromal cells from different sources. Exp Cell Res..

[60] Wagner W, Wein F, Seckinger A, Frankhauser M, Wirkner U, Krause U (2005). Comparative characteristics of mesenchymal stem cells from human bone marrow, adipose tissue, and umbilical cord blood. Exp Hematol..

[61] Wang A, Du Y, He Q, Zhou C (2013). A quantitative system for discriminating induced pluripotent stem cells, embryonic stem cells and somatic cells. PLoS One..

[62] Baer PC, Kuci S, Krause M, Kuci Z, Zielen S, Geiger H (2013). Comprehensive phenotypic characterization of human adipose-derived stromal/stem cells and their subsets by a high throughout technology. Stem Cells Dev..

[63] Main H, Radenkovic J, Kosobrodova E, McKenzie D, Bilek M, Lendahl U (2014). Cell surface antigen profiling using a novel type of antibody array immobilized to plasma ion-implanted polycarbonate. Cell Mol Life Sci..

[64] Hughes OR, Stewart R, Dimmick I, Jones EA (2009). A critical appraisal of factors affecting the accuracy of results obtained when using flow cytometry in stem cell investigations: where do you put your gates?. Cytometry Part A..

[65] Tormin A, Brune JC, Olsson E, Valcich J, Neuman U, Olofsson T (2009). Characterization of bone marrow-derived mesenchymal stromal cells (MSC) based on gene expression profiling of functionally defined MSC subsets. Cytotherapy..

[66] Brown JP, Finley VG, Kuo CK (2014). Embryonic mechanical and soluble cues regulate tendon progenitor cell gene expression as a function of developmental stage and anatomical origin. J Biomech..

[67] Havis E, Bonnin MA, Olivera-Martinez I, Nazaret N, Ruggiu M, Weibel J (2014). Transcriptomic analysis of mouse limb tendon cells during development. Development..

[68] Yin Z, Chen X, Chen JL, Shen WL, Hieu Nguyen TM, Gao L (2010). The regulation of tendon stem cell differentiation by the alignment of nanofibers. Biomaterials..

[69] Yin Z, Chen X, Zhu T, Hu JJ, Song HX, Shen WL (2013). The effect of decellularized matrices on human tendon stem/progenitor cell differentiation and tendon repair. Acta Biomater..

[70] Rui Y, Guo Y, Lin Y, Ma L, Cheng X, Chen H (2013). Experiment of bone morphogenetic protein 2 induced chondrogenic differentiation of human Achilles tendon-derived stem cells in vitro. Zhongguo Xiu Fu Chong Jian Wai Ke Za Zhi.

[71] Stanco D, Vigano M, Perucca Orfei C, Di Giancamillo A, Peretti GM, Lanfranchi L, et al. Multidifferentiation potential of human mesenchymal stem cells from adipose tissue and hamstring tendons for musculoskeletal cell-based therapy. Regen Med. 2015. doi:10.2217/rme.14.92. [Epub ahead of print] 10.2217/rme.14.92 25565145

[72] Zhang J, Wang JH (2012). BMP-2 mediates PGE(2)-induced reduction of proliferation and osteogenic differentiation of human stem cells. J Orthop Res..

[73] Zhang J, Keenan C, Wang JH (2013). The effects of dexamethasone on human patellar tendon stem cells: implications for dexamethasone treatment of tendon injury. J Orthop Res..

[74] Zhang J, Li B, Wang JH (2011). The role of engineered tendon matrix in the stemness of tendon stem cells in vitro and the promotion of tendon-like tissue formation in vivo. Biomaterials..

[75] Zhang J, Wang JH (2014). Prostaglandin E2 (PGE2) exerts biphasic effects on human tendon stem cells. PLoS One..

[76] Tsai CC, Huang TF, Ma HL, Chiang ER, Hung SC (2013). Isolation of mesenchymal stem cells from shoulder rotator cuff: a potential source for muscle and tendon repair. Cell Transplant..

[77] Zhang J, Wang JH (2013). The effects of mechanical loading on tendons – an in vivo and in vitro model study. PLoS One..

[78] Chen L, Dong SW, Liu JP, Tao X, Tang KL, Xu JZ (2012). Synergy of tendon stem cells and platelet-rich plasma in tendon healing. J Orthop Res..

[79] Chen L, Liu JP, Tang KL, Wang Q, Wang GD, Cai XH (2014). Tendon derived stem cells promote platelet-rich plasma healing in collagenase-induced rat Achilles tendinopathy. Cell Physiol Biochem..

[80] Rui YF, Lui PPY, Li G, Fu SC, Lee YW, Chan KM (2010). Isolation and characterization of multi-potent rat tendon-derived stem cells. Tissue Eng Part A..

[81] Liu J, Chen L, Tao X, Tang K (2013). Phosphoinositide 3-kinase/Akt signaling is essential for prostaglandin E2-induced osteogenic differentiation of rat tendon stem cells. Biochem Biophys Res Commun..

[82] Rui YF, Lui PP, Wong YM, Tan Q, Chan KM (2013). Altered fate of tendon-derived stem cells isolated from a failed tendon-healing animal model of tendinopathy. Stem Cells Dev..

[83] Shi Y, Fu Y, Tong W, Geng Y, Lui PP, Tang T (2012). Uniaxial mechanical tension promoted osteogenic differentiation of rat tendon-derived stem cells (rTDSCs) via the Wnt5a-RhoA pathway. J Cell Biochem..

[84] Holladay C, Abbah SA, O’Dowd C, Pandit A, Zeugolis DI. Preferential tendon stem cell response to growth factor supplementation. J Tissue Eng Regen Med. 2014. doi:10.1002/term.1852. [Epub ahead of print] 10.1002/term.1852 24474722

[85] Lui PPY, Kong SK, Lau PM, Wong YM, Lee YW, Tan C (2014). Immunogenicity and escape mechanisms of allogeneic tendon-derived stem cells (TDSCs). Tissue Eng Part A..

[86] Zhang J, Pan T, Liu Y, Wang JH (2010). Mouse treadmill running enhances tendons by expanding the pool of tendon stem cells (TSCs) and TSC-related cellular production of collagen. J Orthop Res..

[87] Zhang J, Wang JH (2010). Platelet-rich plasma releasate promotes differentiation of tendon stem cells into active tenocytes. Am J Sports Med..

[88] Yang Y, Zhang J, Qian Y, Dong S, Huang H, Boada FE (2013). Superparamagnetic iron oxide is suitable to label tendon stem cells and track them in vivo with MR imaging. Ann Biomed Eng..

[89] Zhang J, Wang JH (2014). PRP treatment effects on degenerative tendinopathy – an in vitro model study. Muscles Ligaments Tendons J..

[90] Shen W, Chen J, Yin Z, Chen X, Liu H, Heng BC (2012). Allogenous tendon stem/progenitor cells in silk scaffold for functional shoulder repair. Cell Transplant..

