# Supplementary material for: Markers for the identification of tendon-derived stem cells in vitro and tendon stem cells in situ – update and future development
Source: Stem Cell Res Ther. 2015 Jun 2;6(1):106. doi: 10.1186/s13287-015-0097-y (PMC4451873; doi:10.1186/s13287-015-0097-y)
Supplement: Additional file 1: — Contains Table S1 and Table S2 presenting protein expression of positive and negative markers in TDSCs isolated from different tendons in different species. [file 13287_2015_97_MOESM1_ESM.doc]

**Additional File 1. Protein expression of positive and negative markers in TDSCs isolated from different tendons in different species.**

**Table S1**

| **Reference** | **Species** | **Tendon** | ***NS** | ***Oct-4** | **SSEA-1** | **SSEA-4** | ***Nanog** | ***c-Myc** | ***Sox 2** | **TRA-**  **1-60** | **Sca-1** | ***α-**  **SMA** | **Musashi**  **-1^a^** | **MHCI** | ***Fas** | ***FasL** | **CD9** | **CD13** | **CD29** | **CD44** | **CD49e** | **CD71** | **CD73** | **CD90** | **CD90.1** | **CD90.2** | **CD105** | **CD146** | **CD147** | **CD166** | **Stro-1** | ***Nestin** | **P75** | ***Vimen-**  **Tin** | ***Snail** | ***Sox 10** |
| --- | --- | --- | --- | --- | --- | --- | --- | --- | --- | --- | --- | --- | --- | --- | --- | --- | --- | --- | --- | --- | --- | --- | --- | --- | --- | --- | --- | --- | --- | --- | --- | --- | --- | --- | --- | --- |
| [27,68-70] | Human | Achilles tendon |  | **+** |  | **+** |  |  |  |  |  |  | **+** |  |  |  |  |  |  | **+** |  |  | **+** | **+** |  |  | **+** | **+/-** |  | **+** | **+** |  |  |  |  |  |
| [3,38,71] | Human | Hamstring tendon |  |  |  |  |  |  |  |  |  |  |  |  |  |  |  | **+** | **+** | **+** |  | **-** | **+** | **+** |  |  | **+** | **+** |  | **+** | **+** |  |  |  |  |  |
| [39,40,  72-75] | Human | Patellar tendon | **+** | **+** | **+** | **+** | **+** |  |  |  |  |  |  |  |  |  |  |  |  | **+** |  |  | **+** | **+** |  |  | **+** | **-** |  |  | **+/-** |  |  |  |  |  |
| [4,5,11,76] | Human | Rotator cuff tendon |  |  | **-** | **+/-** |  |  |  |  |  | **+** |  |  |  |  | **+** | **+** | **+** | **+** |  | **+** | **+** | **+** |  |  | **+/-** | **+** | **+** | **+/-** | **+/-** |  | **-** |  |  |  |
| [30,35,77] | Mouse | Achilles tendon | **+** | **+** | **+** |  |  |  |  |  | **+** |  |  |  |  |  |  |  | **+** | **+** | **+** |  |  |  |  | **+** | **+** |  |  |  |  |  |  |  |  |  |
| [3,77] | Mouse | Patellar tendon | **+** | **+** | **+** |  |  |  |  |  | **+** |  |  |  |  |  |  |  |  | **+** |  |  |  |  |  | **+** |  |  |  |  |  |  |  |  |  |  |
| [18] | Mouse | Tail tendon |  |  |  |  | **+** |  |  |  | **+** |  |  |  |  |  |  |  |  | **+** |  |  | **+** |  |  | **+** | **+** | **+** |  |  |  | **+** |  |  |  |  |
| [78,79] | Rat | Achilles tendon |  |  |  |  |  |  |  |  |  |  |  |  |  |  |  |  |  | **+** |  |  |  | **+** |  |  |  |  |  |  |  |  |  |  |  |  |
| [80,81] | Rat | Flexor tendon | **+** | **+** |  | **+** |  |  |  |  |  |  |  |  |  |  |  |  |  | **+** |  |  |  | **+** |  |  |  |  |  |  |  |  |  |  |  |  |
| [16,22,24, 25,33,44, 82-85] | Rat | Patellar tendon | **+** | **+** | **+** | **+** | **+** |  | **+** |  |  |  |  | **+** | **+** | **+** |  |  | **#+** | **+** |  |  | **+** | **#+** | **+** |  |  | **+** |  |  |  |  | **#+** | **#+** | **#+** | **#+** |
| [26,86] | Rabbit | Achilles tendon | **+/-** | **+** |  | **+** |  |  |  |  |  |  |  |  |  |  |  |  |  |  |  |  |  |  |  |  |  |  |  |  |  |  |  |  |  |  |
| [21,26,74, 86-89] | Rabbit | Patellar tendon | **+/-** | **+** | **+** | **+** | **+** |  |  |  |  |  |  |  |  |  |  |  |  |  |  |  |  |  |  |  |  |  |  |  |  |  |  |  |  |  |
| [90] | Rabbit | Rotator cuff tendon |  |  |  |  |  |  |  |  |  |  |  |  |  |  |  |  |  |  |  |  |  |  |  |  |  |  |  |  |  |  |  |  |  |  |
| [23] | Horse | Superficial digital flexor tendon |  | **+** |  | **+** |  | **+** |  | **+/-** |  |  |  |  |  |  |  |  |  |  |  |  |  |  |  |  |  |  |  |  |  |  |  |  |  |  |

**Table S2**

| **Reference** | **Species** | **Tendon** | **CD2** | **CD3** | **CD11b** | **CD14** | **CD16** | **CD18** | **CD19** | **CD31** | **CD34** | **CD45** | **CD54** | **CD56** | **CD80** | **CD86** | **CD106** | **CD117** | **CD123** | **CD133** | **CD144** | **CD177** | **CD235a** | **Flk-1 (VEGFR-2)** | **MHC-DR** | **Gr-1** |
| --- | --- | --- | --- | --- | --- | --- | --- | --- | --- | --- | --- | --- | --- | --- | --- | --- | --- | --- | --- | --- | --- | --- | --- | --- | --- | --- |
| [27,68-70] | Human | Achilles tendon |  |  |  |  |  | **-** | **-** |  | **-** | **-** |  |  |  |  |  |  |  |  |  |  |  |  | **-** |  |
| [3,38,71] | Human | Hamstring tendon |  |  |  | **-** |  | **-** | **-** | **-** | **-** | **-** | **+** |  |  |  | **-** |  |  |  |  | **-** |  |  | **-** |  |
| [39,40, 72-75] | Human | Patellar tendon |  |  |  |  |  |  |  |  | **-** | **-** |  |  |  |  |  |  |  |  |  |  |  |  |  |  |
| [4,5,11,76] | Human | Rotator cuff tendon | **-** | **-** | **-** | **-** | **-** | **-** | **-** | **-** | **-** | **-** |  | **-** |  |  | **+/-** | **-** | **-** | **-** |  |  | **-** | **-** | **-** |  |
| [30,35,77] | Mouse | Achilles tendon |  |  | **-** |  |  | **-** |  |  | **+/-** | **-** |  |  |  |  |  |  |  | **-** |  |  |  |  |  | **-** |
| [3,77] | Mouse | Patellar tendon |  |  |  |  |  | **-** |  |  | **-** | **-** |  |  |  |  |  |  |  |  | **-** | **-** |  | **-** |  |  |
| [18] | Mouse | Tail tendon |  |  |  |  |  |  |  |  |  |  |  |  |  |  |  |  |  |  |  |  |  |  |  |  |
| [78,79] | Rat | Achilles tendon |  |  |  |  |  |  |  |  | **-** | **-** |  |  |  |  |  |  |  |  |  |  |  |  |  |  |
| [80,81] | Rat | Flexor tendon |  |  |  |  |  |  |  | **-** | **-** |  |  |  |  |  |  |  |  |  |  |  |  |  |  |  |
| [16,22,24, 25,33,44, 82-85] | Rat | Patellar tendon |  |  | **-** |  |  |  |  | **-** | **#-** | **#-** | **#-** |  | **-** | **-** | **-** |  |  |  |  |  |  |  | **-** |  |
| [26,86] | Rabbit | Achilles tendon |  |  |  |  |  |  |  |  |  |  |  |  |  |  |  |  |  |  |  |  |  |  |  |  |
| [21,26,74, 86-89] | Rabbit | Patellar tendon |  |  |  |  |  |  |  |  |  |  |  |  |  |  |  |  |  |  |  |  |  |  |  |  |
| [90] | Rabbit | Rotator cuff tendon |  |  |  |  |  |  |  |  |  |  |  |  |  |  |  |  |  |  |  |  |  |  | **-** |  |
| [23] | Horse | Superficial digital flexor tendon |  |  |  |  |  |  |  |  | **-** |  |  |  |  |  |  |  |  |  |  |  |  |  |  |  |

+, positive expression; –, null expression; +/–, unclear due to low expression level or inconsistent findings in different studies. *Intracellular expression; # includes study [33] in which cells were cultured in neural crest stem cell medium. The markers were evaluated either by flow cytometry or immunofluorescent staining.
